# Current Approaches in the Treatment of Relapsed and Refractory Acute Myeloid Leukemia

**DOI:** 10.3390/jcm4040665

**Published:** 2015-04-10

**Authors:** Nestor R. Ramos, Clifton C. Mo, Judith E. Karp, Christopher S. Hourigan

**Affiliations:** 1 Myeloid Malignancies Section, Hematology Branch, National Heart, Lung and Blood Institute, National Institutes of Health, Bethesda, MD 20892-1583, USA; E-Mail: nestor.r.ramos.mil@mail.mil; 2 Department of Hematology-Oncology, John P. Murtha Cancer Center, Walter Reed National Military Medical Center, Bethesda, MD 20889, USA; E-Mail: clifton.c.mo.mil@mail.mil; 3 Division of Hematologic Malignancies, Sidney Kimmel Comprehensive Cancer Center, Johns Hopkins School of Medicine, Baltimore, MD 21205, USA; E-Mail: jkarp2@jhmi.edu

**Keywords:** salvage therapy, leukemia, neoplasm metastasis, AML, relapse

## Abstract

The limited sensitivity of the historical treatment response criteria for acute myeloid leukemia (AML) has resulted in a different paradigm for treatment compared with most other cancers presenting with widely disseminated disease. Initial cytotoxic induction chemotherapy is often able to reduce tumor burden to a level sufficient to meet the current criteria for “complete” remission. Nevertheless, most AML patients ultimately die from their disease, most commonly as clinically evident relapsed AML. Despite a variety of available salvage therapy options, prognosis in patients with relapsed or refractory AML is generally poor. In this review, we outline the commonly utilized salvage cytotoxic therapy interventions and then highlight novel investigational efforts currently in clinical trials using both pathway-targeted agents and immunotherapy based approaches. We conclude that there is no current standard of care for adult relapsed or refractory AML other than offering referral to an appropriate clinical trial.

## 1. Introduction

Approximately 20,000 patients will be diagnosed with acute myeloid leukemia (AML) with greater than 10,000 AML patient deaths in the United States during 2015 [1]. While complete response rates can be as high as 80% in patients undergoing initial induction cytotoxic chemotherapy, the majority of AML patients will ultimately be diagnosed with relapsed or refractory disease [2,3]. Patients with relapsed or refractory AML (RR-AML) have, in general, a poor prognosis [4,5]. While several factors have been associated with worse outcomes at relapse, including unfavorable cytogenetics at diagnosis, duration of first complete response (CR) less than 12 months, older age, and prior history of hematopoietic stem cell transplant (HSCT) [5,6,7] even a patient without such adverse factors faces a formidable challenge in achieving and maintaining a second remission.

It is now clear that AML is a diagnosis encompassing a wide range of myeloid malignancies with heterogeneous genetic etiology [8]. It is also clear that AML is an oligoclonal disease even within a single patient, such that the predominant clone at initial presentation is not necessarily identical to the clone ultimately responsible for clinical relapse and death [9,10]. AML presenting as clinical relapse may be from three underlying sources: (1) chemo-sensitive disease that was only partially treated and returns, perhaps with additional mutations; (2) a subclone, derived from the same founder clone as the predominant clone, initially present at low frequency but given a clonal advantage during treatment due to decreased chemotherapy sensitivity; and (3) *de novo* generation of AML due to toxicity from treatment. Experimental evidence to date suggests the first two mechanisms are the most common [9,10], although the last may be operable in late relapses that occur three or more years after achieving initial CR.

While a variety of different treatment regimens have been studied in an effort to improve outcomes of patients with RR-AML, there appears to be no single superior approach. We therefore believe that the only current standard of care for a patient with relapsed AML is to offer enrollment in a clinical trial [11]. This article will review combinatorial chemotherapy regimens (both traditional and investigational) used in the AML (non-APL) salvage setting (Table 1), and will then discuss novel single agent approaches, including targeted small molecule drugs for known AML mutations and/or pathways (Table 2), as well as immunomodulatory drugs and antibody-based, vaccine-based, and adoptive cellular immunotherapies (Table 3).

## 2. Cytotoxic Chemotherapy

While most patients able to undergo initial cytotoxic induction therapy will initially achieve a CR, the most common cause of death is subsequent relapse of disease. Treatment options in this setting include aggressive therapy geared towards providing a bridge to an allogeneic hematopoietic stem cell transplantation (alloHSCT), which is often considered the only potentially curative option for patients with RR-AML, or best supportive care with palliative intent in patients who are not candidates for an aggressive approach. Patients must have a suitable donor, good performance status, minimal comorbidities, and generally should be in at least a CR prior to undergoing alloHSCT.

**Table 1:** Conventional and novel cytotoxic salvage chemotherapy regimens utilized in patients with relapsed/refractory acute myeloid leukemia (AML).

Regimen	Agents	CR	TRM or 30 Day Mortality	Reference
HIDAC	Cytarabine 3 g/m^2^ every 12 h days 1–6	32%–47%	12%–15%	[12,13]
FLAG FLAG-IDA	Fludarabine 30 mg/m^2^ days 1–5	48%–55%	10%–11%	[14,15]
	Cytarabine 2 g/m^2^ days 1–5			
	G-CSF 5 mcg/kg day 0 until ANC recovery			
	Fludarabine 30 mg/m^2^ days 1–5	63%	17%	[16]
	Cytarabine 2 g/m^2^ days 1–5			
	G-CSF 300 mcg day 0 until ANC recovery			
	Idarubicin 8 mg/m^2^ days 1–3			
FLA	Fludarabine 30 mg/m^2^ days 1–5	61%	7%	[17]
	Cytarabine 2 g/m^2^ days 1–5			
CLAG CLAG-M	Cladribine 5 mg/m^2^ days 2–6	38%–50%	0%–17%	[18,19,20]
	Cytarabine 2 g/m^2^ days 2–6			
	G-CSF 300 mcg days 1–6			
	Cladribine 5 mg/m^2^ days 1–5	50%–58% (53% after first course)	0%–7%	[18,21]
	Cytarabine 2 g/m^2^ days 1–5			
	G-CSF 300 mcg days 0–5			
	Mitoxantrone 10 mg/m^2^ days 1–3			
MEC	Mitoxantrone 6 mg/m^2^ days 1–6	59%–66%	3%–6%	[22,23]
	Etoposide 80 mg/m^2^ days 1–6			
	Cytarabine 1 g/m^2^ days 1–6			
	Mitoxantrone 8 mg/m^2^ days 1–5	18%–24%	7%–11%	[20,24,25]
	Etoposide 100 mg/m^2^ days 1–5			
	Cytarabine 1 mg/m^2^ days 1–5			
MEC/Decitabine	Decitabine 20 mg/m^2^ days 1–10	30% (CR + CRp + CRi = 50%)	20%	[26]
	Mitoxantrone 8 mg/m^2^ days 16–20			
	Etoposide 100 mg/m^2^ days 16–20			
	Cytarabine 1 mg/m^2^ days 16–20			
EMA-86	Mitoxantrone 12 mg/m^2^ days 1–3	60%	11%	[27]
	Cytarabine 500 mg/m^2^ CI days 1–3 & 8–10			
	Etoposide 200 mg/m^2^ CI days 8–10			
MAV	Mitoxantrone 10 mg/m^2^ days 4–8	58%	11%	[28]
	Cytarabine 100 mg/m^2^ CI days 1–8			
	Etoposide 100–120 mg/m^2^ days 4–8			
FLAD	Fludarabine 30 mg/m^2^ days 1–3	53%	7.5%	[29]
	Cytarabine 2 g/m^2^ days 1–3			
	Liposomal daunorubicin 100 mg/m^2^ days 1–3			
FLAM	Flavopiridol 50 mg/m^2^ days 1–3	28%–43%	5%–28%	[30,31]
	Cytarabine 2 g/m^2^/72 h starting day 6			
	Mitoxantrone 40 mg/m^2^ day 9			
Hybrid FLAM	Flavopiridol 30mg/m^2^ bolus, 60 mg/m^2^ over 4 h days 1–3	40%	9%	[32]
	Cytarabine 2 g/m^2^/72 h starting day 6			
	Mitoxantrone 40 mg/m^2^ day 9			
Clofarabine Cytarabine	Clofarabine 40 mg/m^2^ days 2–6	28%–51%	6.2%–13%	[33]
	Cytarabine 1 g/m^2^ days 1–5			
	Clofarabine 40 mg/m^2^ days 1–5; Cytarabine 1 g/m^2^ days 1–5			[34,35]
	Clofarabine 22.5 mg/m^2^ days 1–5; Cytarabine 1 g/m^2^ days 1–5			
GCLAC	Clofarabine 25 mg/m^2^ days 1–5; Cytarabine 2 g/m^2^ days 1–5; G-CSF 5 mcg/kg day 0 until ANC recovery	46%, (CR + CRp 61%)	13%	[36,37]
HAA	Homoharringtonine 4 mg/m^2^ days 1–3	76%–80%	0%	[38]
	Cytarabine 150 mg/m^2^ days 1–7			
	Aclarubicin 12 mg/m^2^ days 1–7			
CPX 351	CPX 351 101 units/m^2^ days 1, 3, and 5	23%–37% (CR + CRi = 49%)	7%–13%	[39]
	CPX 351 100 units/m^2^ days 1, 3, 5 (first induction) and days 1 and 3 (second induction and consolidation)			[40]

Abbreviations: complete response (CR), complete response with incomplete platelet recovery (CRp), complete response with incomplete blood count recovery (CRi), treatment-related mortality (TRM), High-dose arabinoside cytarabine (HIDAC), granulocyte colony stimulating factor (G-CSF), continuous infusion (CI), cyclin-dependent kinase (CDK).

**Table 2:** Targeted agents under evaluation for treatment of patients with relapsed/refractory AML (RR-AML). *Ongoing clinical trials (either enrolling new patients with RR-AML or active but no longer enrolling patients).*

Agent	Mechanism of Action	Ongoing Clinical Trial	Reference
Ruxolitinib	JAK1 and JAK2 inhibitor	NCT02257138, NCT00674479, NCT01251965	[41,42]
Rapamycin	mTOR inhibitor	NCT01184898, NCT01869114, NCT00634244, NCT02109744	[43,44]
Everolimus	mTOR inhibitor	NCT00819546	[45]
Tosedostat	Aminopeptidase activity inhibitor	NCT01636609	[46,47]
Vorinostat	Histone deacetylase inhibitor	NCT01130506, NCT01534260, NCT01550224, NCT01617226, NCT02083250	[48,49,50]
AG-120	IDH1 inhibitor	NCT02074839	NCT02074839
AG-221	IDH2 inhibitor	NCT01915498	[51]
Elacytarabine	Elaidic acid ester of cytarabine	No active studies found	[52]
Vosaroxin	Anticancer quinolone derivative	NCT01191801	[53,54]
Pravastatin	HMG-CoA reductase inhibitor	NCT00840177	[55,56]
Bortezomib	Proteasome inhibitor	NCT01174888, NCT01127009, NCT01736943, NCT01861314, NCT01534260, NCT01075425, NCT00410423	[57]
Lenalidomide	Immunomodulatory agent	NCT01681537, NCT01904643, NCT01629082, NCT01132586, NCT01246622, NCT01743859, NCT01016600, NCT00466895, NCT01615042	[58]
CPI-613	Lipoate derivative	NCT01768897	[59]
ABT-199	BCL-2 inhibitor	NCT01994837	[60]
Erismodegib	Hedgehog inhibitor	NCT02129101	NCT02129101
PF-04449913	Hedgehog inhibitor	NCT02038777	NCT02038777

Source: www.clinicaltrials.gov.

**Table 3:** Immunotherapeutic agents under evaluation for treatment of patients with relapsed/refractory AML.

Agent	Mechanism of Action	Ongoing Clinical Trial	Reference
Gemtuzumab ozogamicin	Conjugated Antibody targeting CD33	NCT01869803, NCT00766116, NCT02221310	[61,62,63]
SGN-CD33A	Conjugated Antibody targeting CD33	NCT01902329	[64,65]
Lintuzumab	Unconjugated Antibody targeting CD33	No active studies found	[66]
CSL362	Unconjugated Antibody targeting CD123	No active studies found	[67,68]
AMG330	Bispecific T-cell Engaging Antibody targeting CD33 and CD3	No active studies found	[69,70]
MGD006	Dual Affinity Re-Targeting Antibody targeting CD123 and CD3	NCT02152956	[71]
CD16x33 BiKE	Bispecific Killer Cell Engager Antibody against CD16 and CD33	No active studies found	[72]
CART33	Chimeric Antigen Receptor-Transduced T Cells targeting CD33	NCT01864902	[73]
CART123	Chimeric Antigen Receptor-Transduced T Cells targeting CD123	NCT02159495	[74,75]
WT1 peptide vaccine	Vaccine targeting WT1	NCT00965224	[76,77]
WT1-specific CD8(+) T-cell infusion	Adoptive Cell Transfer	NCT01640301	[78,79,80]
Haploidentical NK cell infusion	Adoptive Cell Transfer	NCT01947322, NCT01385423, NCT01370213, NCT00303667, NCT01621477, NCT00526292, NCT00789776, NCT02259348, NCT01795378, NCT01898793, NCT01386619	[81]
AlloHSCT	Adoptive Cell Transfer	More than 40 active clinical trials identified	[82,83,84,85]
Donor lymphocyte infusion (post alloHCT)	Adoptive Cell Transfer	NCT01758367, NCT01390311, NCT00068718, NCT01523223, NCT01760655, NCT00534118, NCT00005799, NCT00448357	[86,87,88,89]

Source: www.clinicaltrials.gov.

Most commonly used induction regimens involve a combination of infusional cytarabine, an anthracycline, and perhaps an additional agent. Cytarabine, a deoxycytidine analog that is actively metabolized to arabinofuranosylcytidine triphosphate (ara-CTP), serves as the backbone for many commonly utilized salvage regimens, demonstrating efficacy in relapsed AML both as monotherapy when given in high doses (HIDAC), and in combination with other therapeutic agents [12,13]. In general, patients with a good performance status who have not yet received HIDAC can receive this treatment regimen in the salvage setting with or without an anthracycline, as per NCCN guidelines [90]. Patients who suffer a late relapse, especially one greater than 18 months from initial CR (CR1), can sometimes achieve a second CR (CR2) by retreatment with the same initial induction regimen [90]. With reported CR2 rates of only 32% to 47%, however, HIDAC monotherapy is far from an acceptable standard of care for relapsed disease [12,13].

FLA/FLAG—Fludarabine is a purine analog that acts by inhibiting ribonucleotide reductase (RNR), which augments the rate of synthesis of ara-CTP in circulating AML blasts when infused prior to cytarabine [91]. It has been studied in relapsed AML in combination with high-dose cytarabine (FLA), with or without granulocyte-colony stimulating factor (G-CSF), with reported CR rates ranging from 46% to 63% [14,15,16] (Table 1). Idarubicin, an anthracycline, is frequently added to FLAG and despite a lack of evidence of improved outcome is a reasonable option in select patients [16]. Notably, FLA did not produce superior CR rates in the salvage setting when compared to standard induction chemotherapy (cytarabine, daunorubicin, and etoposide) in the randomized MRC AML-HR trial (CR rates: 61% FLA *versus* 63% ADE, *p* = 0.8), and in fact survival at 4 years was significantly inferior using this approach (16% *versus* 27%, *p* = 0.05) [17]. Likewise, the addition of G-CSF to FLA in this trial failed to demonstrate improvement in outcomes over FLA alone (CR rates: 58% G-CSF *versus* 61% no G-CSF, *p* = 0.7). Nevertheless, FLAG ± idarubicin remains an accepted alternative to HIDAC monotherapy in relapsed AML.

CLAG/CLAG-M—Cladribine is another RNR-inhibiting purine analogue that was found to yield synergistic effects on inhibition of cell proliferation, induction of apoptosis, and disruption of mitochondrial membrane potential when combined with cytarabine [92]. It has been associated with CR rates ranging from 38% to 58% when combined with high-dose cytarabine and G-CSF (CLAG) in the relapsed setting [18,19,20,21] (Table 1), with the highest rates observed in those treated with mitoxantrone in addition to CLAG (CLAG-M) [21].

MEC/EMA-86/MAV—Combinations of mitoxantrone, etoposide and cytarabine have been extensively evaluated in relapsed AML with multiple variations in the dose and schedule (MEC, EMA-86, MAV), resulting in CR rates between 18% and 66% [20,22,23,24,25,27,28,93] (Table 1) with the highest rates seen when given as timed-sequential therapy [27,93]. A single-institution retrospective review by Price *et al.* evaluated 162 patients with RR-AML treated with CLAG *versus* MEC and found overall CR rates of 37.9% *versus* 23.8% (*p* = 0.05), with a median follow up of 20.3 months [20]. Although limited by the retrospective nature of the study, a possible superiority of CLAG is suggested. Notably, the addition of sirolimus to MEC was evaluated in an arm of the E1906 trial, a phase II study among patients with RR-AML, but closed to accrual early with 15% responses [30]. An ongoing phase II study (NCT01729845) is currently evaluating the effect of pre-treatment “priming” with decitabine, a hypomethylating agent, prior to MEC after a phase I study found a CR rate of 30% (9 of 30 patients; CR + CRp + CRi = 50%) with a treatment related mortality (TRM) of 20% [26].

GCLAC—Clofarabine is a second-generation deoxyadenosine analog that is characterized by high resistance to phosphorolytic cleavage by bacterial purine nucleoside phosphorylase, potent inhibition of DNA synthesis, prolonged retention of clofarabine triphosphate in leukemic blasts [34] and, similar to fludarabine and cladribine, can inhibit RNR reductase and increase the intracellular concentration of ara-CTP when administered prior to cytarabine [94]. Studies evaluating clofarabine with intermediate-dose cytarabine (1 gm/m^2^) have found CR rates 35%–51% [33,34,35] (Table 1). GCLAC is a regimen containing clofarabine, high dose cytarabine (2 g/m^2^), and G-CSF that has resulted in comparable CR rates (46% in a phase I/II study) [36] (Table 1). A retrospective study comparing 50 patients who received GCLAC to 101 patients who received FLAG or FLA demonstrated a superior CR rate for patients who received GCLAC, with an odds ratio of 9.57 (*p* < 0.0001) [37]. GCLAC also demonstrated impressive efficacy as initial induction therapy with an overall CR rate of 76% (CR + CRp = 82%) in a recently reported multicenter trial [95]. Further studies will be necessary to determine conclusively if GCLAC is superior to other approaches in RR-AML.

FLAD—Liposomal daunorubicin has been found to be at least as effective as free (non-liposomal) daunorubicin in leukemic cells and could have decreased toxicity [96,97]. This agent was evaluated in combination with cytarabine and fludarabine (FLAD), with an overall CR rate of 53% (CR in 73% of relapsed AML subjects *versus* 0% of refractory AML subjects) among 41 patients with RR-AML (including two patients with CML in myeloid blast crisis) [29]. Fifty-eight percent of patients who achieved CR were able to proceed to alloHSCT, which raises the possibility of this regimen being utilized as a bridge to transplant.

FLAM—Flavopiridol, a synthetic flavone derivative initially isolated from the stem bark of the Indian tree (*Dysoxylum binectariferum*), has properties as a cyclin-dependent kinase inhibitor and has been evaluated as timed sequential therapy in combination with cytarabine and mitoxantrone (FLAM) with a CR rate of 42.5% in a phase II study of 47 patients with RR-AML, including a CR rate of 75% in those with relapsed AML compared to only 9% in those with primary or multiply refractory disease [31] (Table 1). A phase I trial evaluating a “Hybrid FLAM” regimen, which administers Flavopiridol as a 30-min bolus followed by 4-h infusion, found a CR rate for relapsed AML patients of 92% (11 of 12 patients) at the maximum tolerated dose and 31% for primary refractory patients (5 of 16 patients) [32]. However, a randomized phase II study found an overall CR rate of 28% (six patients achieved CR, four patients CRi) among 36 patients, including patients with primary refractory AML and post-HSCT relapse [30]. This study randomized patients to either FLAM, carboplatin-topotecan (14% patients achieved CR or CRi), or sirolimus-MEC. Early FLAM-related mortality was 28% (with 4 of 10 deaths related to overwhelming tumor lysis and cytokine release syndrome from Flavopiridol), predominantly in patients older than 65 years. Thus this regimen holds promise for younger patients (<60–65 y/o) but may be excessively toxic for the older population.

HAA—Homoharringtonine is an alkaloid derived from *Cephalotaxus fortunei* that, when combined with cytarabine and the anthracycline aclarubicin, was shown to induce a CR rate of 76.1% after one cycle of treatment among 46 patients with RR-AML [38] (Table 1). Patients who did not respond or only had a partial response (PR) underwent an additional cycle of HAA, which increased the overall CR rate to 80.4%. In this study, no TRM was documented, but 89.1% of patients developed infections [38].

CPX351—CPX 351 is a bilamellar liposome that encapsulates cytarabine and daunorubicin in an optimally synergistic fixed molar ratio of 5:1 [39]. It was found to induce a CR in 10 of 43 patients (23%) in a phase I study [39] and yielded a CR rate of 49.3% (37% CR + 12.3% CRi) when compared with provider’s-choice of intensive salvage therapy in adults with first relapse of AML (CR + CRi = 40.9%) in a phase II, multicenter, randomized trial [40] (Table 1). A possible clinical benefit for patients treated with CPX 351 was observed in subjects with poor-risk disease, as defined by European Prognostic Index [40].

Vosaroxin—Vosaroxin is a novel anticancer quinolone derivative that intercalates DNA and inhibits topoisomerase II [53]. It was evaluated in combination with cytarabine in a phase III randomized multinational study comprising 711 patients at 124 sites, where the combination was found to improve CR (30.1% *versus* 16.3%, *p* = 0.00001) and median OS (7.5 months *versus* 6.1 months, 2-sided stratified log-rank *p* = 0.02) when compared to the placebo/cytarabine group. Overall survival (OS) benefit was greatest in patients aged 60 years or older (7.1 months *versus* 5 months, *p* = 0.003) and those with early relapse (6.7 months *versus* 5.2 months, *p* = 0.04) [54]. A separate phase Ib/II study evaluating the same combination among patients with RR-AML found a combined CR + CRi rate of 28% [98]. Notably, the CR + CRi rate was 69% for patients whose initial CR lasted greater or equal to 12 months, while patients with initial CR >3 months and <12 months and patients with refractory disease had CR + CRi of 13% and 21%, respectively.

Elacytarabine is an elaidic acid ester of cytarabine that was compared against seven commonly used salvage regimens (investigator’s choice) in a large phase III study but was not found to improve CR or OS rate [52]. Interestingly no significant difference with regards to OS was found among the different salvage treatment regimens.

## 3. Targeted Agents

Increased understanding of AML biology has led to the identification of deregulated pathways that drive blast proliferation. These discoveries have, in turn, given way to the development of agents targeting these molecular pathways.

Cancer Metabolism—Oncogenic mutations affecting two isoforms of the isocitrate dehydrogenase (IDH) enzyme, IDH1 and IDH2, have been reported in approximately 30% of de novo AML and appear to be an unfavorable prognostic factor [99]. AG-221 is an oral, potent, reversible, and selective inhibitor of the mutated IDH2 protein that is currently under evaluation, with early results of an ongoing phase I study revealing objective responses in 6 of 10 patients thus far including three CRs and two CRp (NCT01915498) [51]. An IDH1 inhibitor, AG-120, is also undergoing phase I testing in patients with RR-AML harboring an IDH1 mutation (NCT02074839).

CPI-613 is a lipoate derivative that disrupts mitochondrial metabolism by inhibiting pyruvate dehydrogenase and α-ketogluterate dehydrogenase [100]. When combined with HIDAC and mitoxantrone in a phase I study, 18 patients (50%) achieved a CR or CRi, while 14% of patients died in the first 30 days [59]. Notably, the CR + CRi rate was also 50% among patients 60 years of age or older [59].

Plerixafor—In an effort to disrupt the interaction between leukemic blasts and the bone marrow microenvironment, which is postulated to be an important mediator of chemotherapy resistance [101], plerixafor, a bicyclam that inhibits the CXCR4 chemokine receptor, was evaluated in combination with MEC for the treatment of RR-AML and was found to induce a CR + CRi rate of 46% in a phase I/II study [102]. A follow up study is currently underway evaluating the effect of G-CSF priming in addition to the abovementioned regimen (NCT00906945).

Kinase inhibition—Internal tandem duplication mutations resulting in constitutive activation of the FMS-like tyrosine kinase receptor 3 (FLT3-ITD) are present in approximately 20%–30% of cases of AML, represent a high-risk feature in normal karyotype AML, and are associated with a high risk of relapse following alloHSCT [103]. Pratz *et al.* examined the efficacy of six FLT3-ITD inhibitors for potency against mutant and wild-type FLT3 as well as for cytotoxic effect against a series of primary blast samples and found that the inhibitors could be ranked, from most to least selective against FLT3-ITD, as quizartinib, sorafenib, sunitinib, KW2449, and lestaurtinib [104].

Quizartinib (AC220) has been shown in a phase I trial to be safe and efficacious in patients with both FLT3-ITD negative and positive RR-AML, with most of the responses being PR or CRi and occurring in FLT3-ITD positive patients [105]. Multiple phase II trials for RR-AML have been conducted [106] but to date have only been reported in abstract form [107,108].

In the recently reported SAL-Soraml trial, sorafenib demonstrated safety and improved event free survival compared to placebo when used as an adjunct to standard induction chemotherapy in 267 younger (age <60) patients with newly diagnosed AML, only 17% of whom were FLT3-ITD mutated (3-year event-free survival 40% *versus* 22%, *p* = 0.013) [109]. It also demonstrated safety with early data suggesting a possible reduction in relapse rates when used as maintenance therapy in FLT3-ITD mutated AML patients after alloHSCT [110,111]. Additionally, sorafenib monotherapy exhibited antileukemic efficacy in patients with RR-AML, including patients who relapsed after alloHSCT, in a small retrospective study including 13 patients [112].

Unfortunately, mutations of the FLT3 kinase domain can arise and limit the efficacy of FLT3 inhibitors [113]. The most commonly observed FLT3 mutation occurs at the D835 residue, followed by the F691L [114]. Crenolanib (CP-868,596) is a novel tyrosine kinase inhibitor (TKI) that was originally developed as a selective and potent inhibitor of PDGFR α and β, but also has high affinity for other type III receptor tyrosine kinases such as FLT3. A preclinical study by Zimmerman *et al.* demonstrated that crenolanib has potent activity against FLT3-ITD as well as FLT3 D835 mutations in binding assays and in Ba/F3 cells from a mouse model. Several phase II trials evaluating crenolanib in patients with RR-AML and FLT3 mutations are ongoing (NCT01522469 and NCT01657682).

Another FLT3 inhibitor that has demonstrated inhibitory activity against FLT3-ITD/N676D, FLT3-ITD/F691L, and FLT3-D835Y mutants is G-749, which displayed potent antileukemic activity in bone marrow blasts from AML patients regardless of FLT3 mutation status [115]. AMG 925 is a FLT3/CDK4 dual kinase inhibitor that also demonstrated potent and selective activity *in vivo* in AML tumor models in preclinical studies [116].

JAK inhibition—The Janus kinase—signal transducer and activator of transcription (JAK-STAT) pathway has been found to be dysregulated in AML [117]. Ruxolitinib is a potent, selective JAK1 and JAK2 inhibitor that was found to be overall reasonably well tolerated in a phase I/II study, where 1 of 26 patients with RR-AML achieved a CRp [41]. A previous phase II study, on the other hand, found that 3 of 18 patients with post myeloproliferative disease-AML were able to achieve a CR or CRi [42].

mTOR inhibition—Mammalian target of rapamycin (mTOR) is a serine/threonine kinase involved in the regulation of cell growth and proliferation by translational control of key proteins [43]. Rapamycin is an mTOR inhibitor that has been evaluated as monotherapy, inducing a PR in four of nine patients [43], and in combination with decitabine, achieving a decline in blast percentage in 4 of 13 patients in a phase I trial [44]. Another mTOR inhibitor, RAD001 (everolimus), was evaluated in combination with 7 + 3 chemotherapy (daunorubicin 60 mg/m^2^ d1 to d3, cytarabine 200 mg/m^2^ d1 to d7) in a phase Ib study of 28 patients (age <65) with relapsed AML and produced a CR rate of 68% [45].

Vorinostat—Vorinostat is a histone deacetylase inhibitor that, despite promising data in phase I trials, was found in a phase II study to have a CR rate of only 4.5% in a group of 22 patients with RR-AML (16 patients) and untreated AML (6 patients) [48]. However, when given in combination with azacitidine and gemtuzumab ozogamicin, 42.3% of patients achieved a CR + CRi [49]. Another study evaluating vorinostat in combination with cytarabine and etoposide found a CR rate of 46% at the maximum tolerated dose of vorinostat 200 mg twice a day [50].

Statins—Interestingly, AML blasts frequently overexpress the genes for the low-density lipoprotein (LDL) receptor. Blockade of 3-hydroxy-3-methylglutaryl coenzyme reductase (HMG-CoAR) inhibits cholesterol uptake and synthesis and is believed to sensitize AML cells to cytotoxic therapy [55]. A phase II study evaluating pravastatin, intermediate-dose cytarabine, and idarubicin in patients with relapsed AML found a CR + CRi of 75%, which suggests that this approach may improve efficacy [56]. Notably, a study evaluating pravastatin in addition to idarubicin and cytarabine in patients with *de novo*, untreated AML did not find a significant improvement in CR rates when compared to historical results [118].

Proteasome Inhibition—Bortezomib, a proteasome inhibitor commonly utilized in multiple myeloma, was administered in combination with MEC chemotherapy in a phase I study, where 17 of 33 evaluable patients (52%) achieved either a CR or CRi and TRM was 9% [57]. Several clinical trials are currently evaluating bortezomib, either as monotherapy or in combination to other agents, in patients with RR-AML (Table 2).

Other Pathways—Tosedostat is a novel oral agent that inhibits aminopeptidase activity, resulting in the depletion of cellular amino acid pools selectively in tumor cells [46]. Several studies have evaluated the efficacy of tosedostat monotherapy in RR-AML with CR rates ranging from 10% to 17% [46,47]. It has been observed that, given the pharmacokinetic characteristics and mechanism of action of tosedostat, patients require treatment for at least 4 weeks (and possibly longer) for full therapeutic effect to be reached [47].

ABT-199 is a selective, orally bioavailable small molecule BCL-2 inhibitor that is currently under evaluation in a phase II study with preliminary data revealing a CR + CRi rate of 15.5% among 32 patients [60].

Hedgehog pathway—Aberrant activation of the hedgehog (Hh) pathway was found to be a feature of some CD34-positive myeloid leukemic cells in a preclinical study, where inhibition of Hh signaling was found to induce apoptosis in Hh-responsive CD34 cells [119]. Several studies utilizing Hh inhibitors in RR-AML are currently ongoing.

## 4. Immunotherapy

Unlike conventional chemotherapy, which primarily targets dividing cells, or small molecules that target specific pathways within blasts, immunotherapeutic interventions aim at directing an immune response against tumor cells. Several immunotherapeutic modalities are currently under evaluation and are detailed below and in Table 3.

Antibodies—Two targets in AML have served as the main focus for monoclonal antibody studies: CD123 and CD33. The latter is expressed on blasts in 80%–90% of patients with AML [61]. Gemtuzumab ozogamicin (GO), a CD33-directed antibody linked to the antibiotic cytotoxin calicheamicin, received accelerated approval by the FDA in 2000 but was withdrawn from the U.S. market in 2010 after an interim analysis of SWOG S0106 failed to demonstrate a benefit of adding GO to standard induction chemotherapy, while finding an increased rate of fatal adverse events (5.8% *versus* 0.8%, *p* = 0.002) [62]. Although subsequent studies evaluating GO alone or in combination have produced mixed results in RR-AML, one recent study using fractionated doses of GO combined with standard dose cytarabine found a CR + CRp rate of 75% and 2-year survival of 51% with a low TRM (8.3%) [63]. Further studies will be necessary to determine the safety of GO when administered in fractionated doses.

Other anti-CD33 antibodies include lintuzumab, an unconjugated humanized murine monoclonal antibody that failed to improve CR rates when combined with MEC in a phase III randomized multicenter study [66], and SGN-CD33A, a monoclonal antibody conjugated to a novel synthetic pyrrolobenzodiazepine dimer, which is a potent DNA cross-linking cytotoxin. Following favorable results in a preclinical study [64], an ongoing phase I trial of SGN-CD33A in relapsed AML or patients who decline conventional induction/consolidation therapy has found evidence of antileukemic activity, with 47% of patients achieving blast clearance (CR + CRi + morphologic leukemia-free state), with no maximum tolerated dose identified at this interim analysis (NCT01902329) [65].

CSL362 is a humanized second-generation anti-CD123 antibody with favorable preclinical data [67] that was found to be safe and well tolerated as maintenance therapy in a phase I study of AML patients with CR + CRp and high risk of relapse (NCT01632852) [68]. A phase 2 study of CSL362 is planned.

Bispecific T-cell-engaging (BiTE) antibodies are single-chain antibodies designed to direct cytotoxic T lymphocytes at a predefined surface antigen on tumor cells. AMG 330 is a human BiTE antibody against CD33 and CD3 that has demonstrated potent cytolysis *in vitro* against human AML cells proportional to the level of cell-surface CD33 expression [69], which varied by cytogenetic and molecular subtype [120]. It has also been shown to improve the survival of NOD/SCID mice with leukemia caused by human MOLM-13 AML cells [70]. Meanwhile, a phase I trial is currently underway to further evaluate MGD 006, a humanized Dual Affinity Re-Targetting (DART) bispecific antibody-based molecule directed against CD123 and CD3 (NCT02152956) [71].

Natural Killer (NK) cells are effector lymphocytes of the innate immune system capable of exerting anti-AML activity, as exemplified by its role in the graft-versus-leukemia (GVL) effect after alloHSCT [72]. A humanized bispecific killer cell engager (BiKE) antibody containing binding sites for CD16 and CD33 (CD16x33 BiKE) has been shown to trigger NK cell activation in preclinical studies and could potentially enhance and direct the GVL effect in patients with CD33+ AML after transplant, especially after CMV reactivation [72]. Another study seeking to exploit the anti-AML activity of NK cells has focused on the adoptive transfer of haploidentical NK cells into patients with poor-prognosis AML, where these cells have successfully expanded *in vivo* and induced CR in 5 of 19 patients [81].

Adoptive Cell Therapy—Following favorable ongoing studies in lymphoid malignancies, chimeric antigen receptor-transduced T cells (CART), which are synthetic transmembrane constructs that combine the specificity of antibody target recognition with the potent effector mechanisms of T-cell immunity, have been designed to target CD33 (CART33) and CD123 (CART123) in AML. A study by Wang *et al.*, in which CART33 cells were administered to a patient with relapsed AML, found a temporal decrease in blast ratio from >50% to <6% two weeks after infusion. Unfortunately, the patient then developed cytokine release syndrome requiring a TNF-α inhibitor and subsequently died from disease progression 13 weeks after CART33 infusion [73]. On the other hand, CART123 cells have demonstrated antileukemic activity in animal studies [74,121] and could potentially be superior to CART33 based on their lower toxicity profile against normal hematopoietic cells and identical killing profile against malignant myeloid cells [75]. A phase I study evaluating CART123 in patients with RR-AML is reportedly scheduled to begin soon (NCT02159495).

The Wilms tumor 1 (WT1) gene, a zinc finger transcription factor implicated in leukemogenesis, is overexpressed in 70% of AML patients [78,79] and has been targeted through the adoptive transfer of WT1-specific CD8(+) cytotoxic T-cell clones. In post-transplant patients, these T-cells were found to be safe and able to persist, with evidence of antileukemic activity in some patients [80].

Donor lymphocyte infusion (DLI) can be considered for patients who relapse after undergoing an alloHSCT in an attempt to induce a GVL effect. Generally patients who achieve a CR prior to DLI have a better chance of achieving a durable remission [86,87]. Interestingly, animal studies suggest a possible immunomodulatory effect of azacitidine that might attenuate graft-*versus*-host disease (GVHD) after DLI [88]. A recent phase I study evaluated azacitidine after DLI in eight patients with relapsed AML after alloHSCT and found six CRs and only grade 1 or 2 acute GVHD, which suggests that azacitidine after salvage chemotherapy and DLI is well tolerated and does not appear to hinder neutrophil recovery [89].

Vaccines—A number of studies have looked at vaccination with different leukemia-associated antigens including WT1, PR1, proteinase 3, and RHAMM, with the goal of establishing an immunological response capable of eradicating malignant cells [122]. These studies have generally demonstrated safety and immune correlates but no clinical efficacy. However, a recent phase I/II study of 30 AML patients (3 in PR, 27 in CR with high-risk of relapse) administered a dendritic cell WT1 vaccine in the adjuvant setting and found that 8 of 23 patients with elevated WT1 transcript had a molecular response following vaccination, with five of these eight patients subsequently maintaining remission at a median follow-up of 63 months [76]. This included one patient who was in PR at time of vaccination and is reportedly still in CR more than 5 years after initial diagnosis. A separate phase II study evaluating a multivalent WT1 peptide vaccine in patients with AML in CR is currently underway (NCT01266083).

Allogeneic Hematopoietic Stem Cell Transplant—AlloHSCT is an aggressive intervention that provides patients with RR-AML who have a good performance status and an adequate donor with the best chance of achieving a durable remission [82,123]. Relapsed AML patients entering an alloHSCT in CR have significantly superior OS compared to those with residual detectable disease [82,124]. However, patients with RR-AML who are not in CR prior to undergoing an alloHSCT can still experience improved long-term survival as evidenced by Duval *et al.*, who found an OS of 19% at 3 years among 1673 patients [125]. Patients lacking a suitable matched-related donor have other options for stem cell sources available, including matched unrelated donor, double cord blood units, and haploidentical (haplo) family donor. Notably, haplo-identical donors are often readily available from family members, providing a rapid alternative route to transplantation with acceptable toxicity in relapsed AML patients without a matched related donor option [29,126,127]. Unfortunately a large number of patients with RR-AML are elderly or have less than ideal performance status, which restricts their ability to undergo an alloHSCT with myeloablative conditioning (MAC). Reduced-intensity conditioning (RIC) regimens have been designed to address this problem and, in general, are thought to be associated with decreased TRM at the expense of increased risk of relapse when compared to MAC [83]. A recent retrospective study comparing RIC *versus* MAC alloHSCT among 132 patients aged 35 years or older with AML (including patients in CR1, CR2 or greater, or with refractory disease) found a lower 4-year non-relapse mortality with RIC (13% *versus* 28%, *p* = 0.009), a similar 4-year relapse rate (44% *versus* 33%, *p* = 0.22) and similar overall survival (50% *versus* 43%, *p* = 0.38) [84]. Survival of AML patients relapsing after transplantation is dismal. Response rates for patients who receive intensive salvage therapy after relapse, which offers the best chance for response, is approximately 30% [85]. When post-alloHSCT patients do achieve CR, outcomes have been found to be better with the use of donor cells for consolidation: 2 year OS 55% ± 11% in patients who received either DLI or second HSCT, as compared to 20% ± 10% in patients who only received initial salvage chemotherapy [85]. Azacitidine monotherapy can also be utilized and was found to induce a CR in 15% of patients (CR + PR = 22%) from a total of 204 patients with AML/MDS who relapsed after undergoing an alloHSCT [128].

## 5. Low-Intensity Therapy

Not all patients with RR-AML will be good candidates for the aggressive salvage therapy as described above due to reasons such as poor performance status, comorbidities, or cumulative toxicity from prior therapies. Treatment options in this setting are often palliative in nature and include best supportive care or less aggressive therapies such as hypomethylating agents or low-dose cytarabine (LDAC), which can induce CR rates of around 17% [129].

Hypomethylating agents—The hypomethylating agents 5-azacitidine and decitabine are cytidine analogs that act in part by inhibiting DNA methyltransferases [130] and are increasingly used during induction or salvage therapy in patients who are not good candidates for “aggressive” treatment [90]. A retrospective review encompassing three institutions in France found a CR rate of 21% with 11% PR and an overall survival of 9 months (median OS not reached for responders, 4.5 months OS for non-responders) among 47 patients with RR-AML receiving azacitidine 75 mg/m^2^ days 1–7, while patients were able to proceed with an alloHSCT with reduced intensity conditioning [131]. Decitabine, on the other hand, was found to have a CR rate of 15.7% and a median OS of 177 days among 102 patients with RR-AML in a retrospective study by Ritchie *et al.* [132]. Interestingly, TET2 mutations have recently been proposed as a predictive biomarker of responsiveness to hypomethylating agents in myelodysplastic syndrome (MDS) and low blast count AML [133,134]. It remains to be seen if the identification of TET2 mutations will be useful in patients with AML regardless of blast count.

## 6. Discussion

Most patients who are diagnosed with AML will die of AML. Even within the favorable-risk subgroup of patients, as currently defined by cytogenetic and molecular criteria, many do not enjoy long-term survival. Although standard induction and consolidation chemotherapy regimens are undoubtedly intensive, a fundamental problem in the treatment of AML is not that available therapies are toxic but rather they are not effective enough [135]. Moreover, this therapeutic failure has been masked by the lack of progress in high sensitivity treatment response criteria [136]; a recent review of almost 5000 patients treated on clinical trials and/or in leukemia centers of excellence showed that most patients (mean 79%) treated with curative intent induction chemotherapy had achieved a “complete remission” endpoint as we currently define it, despite a median overall survival of only 20 months for the group [2]. Compounding the difficulty in curing AML is the fact that it is predominantly a disease of older adults who are less likely to be treated intensively [137,138]. While the outcomes for untreated and refractory AML are dire, those for relapsed AML are only slightly better with less than 30% of patients surviving 12 months after relapse [5,7,85].

**Figure 1:**
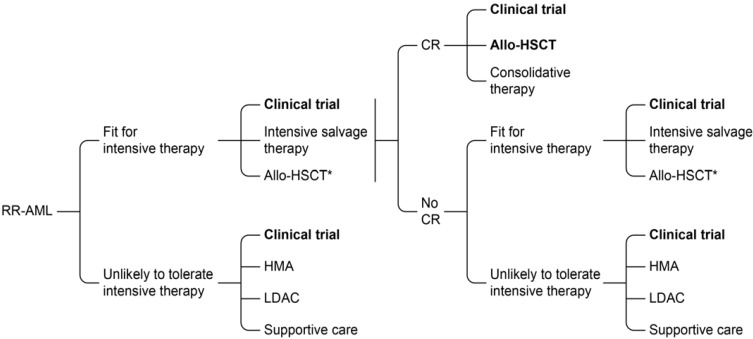
Treatment algorithm for patients with RR-AML in 2015. There is no standard of care for the treatment of relapsed or refractory AML. A clinical trial is always the preferred option. The above algorithm is based on current clinical practice and will hopefully change in coming years due to improvements. In particular the targeted and immunotherapeutic agents detailed in this review may ultimately have utility in (1) initial therapy; (2) as a bridge to, or as a temporizing measure before, allo-HSCT; and/or (3) as part of consolidative therapy. * Achievement of a complete remission (CR) prior to undergoing alloHSCT is associated with best survival and is generally preferred. The survival of patients with residual disease undergoing alloHSCT varies considerably however and this therapy may be a reasonable option in selected patients not in CR [125]. HMA: Hypomethylating agent. LDAC: Low-dose cytosine arabinoside. Allo-HSCT: Allogeneic Hematopoietic Stem Cell Transplant.

In RR-AML patients with poor performance status, prohibitive comorbidities, or those who do not wish to undergo aggressive therapy, options are currently limited to best supportive care or low-intensity therapy with palliative intent. Although patients who are good candidates for aggressive treatment have a significant number of salvage therapy options available to them, none has categorically proven to offer superior outcomes and participation in clinical trials should be encouraged (Figure 1). In general, aggressive salvage therapies are given with the intent of maximally “debulking” the disease burden prior to proceeding with an alloHSCT. Indeed, the depth of remission when entering alloHSCT (as measured by minimal residual disease, or MRD), rather than its chronological setting (CR1 *versus* CR2), may be most predictive of overall survival [124]. Unfortunately, with a median age at diagnosis of 67, many AML patients are poor candidates for traditional alloHSCT due to comorbidity or poor performance status. However, despite a significant non-relapse mortality risk, alloHSCT provides the best chance of achieving a durable response at this time [85,125]. Patients who relapse after alloHSCT have a grim prognosis [85].

AML has historically been approached as a homogeneous diagnostic entity with a resulting “one size fits all” treatment strategy, often resulting in disappointing outcomes. In reality, the acute myeloid leukemias are a heterogeneous group of diseases with distinct molecular and phenotypic characteristics. While factors such as patient age, secondary AML, WBC count at presentation, and cytogenetic and molecular markers all are *associated* with treatment resistance, they offer suboptimal predictive power for the individual patient [2]. Heterogeneity in disease biology explains, in part, the unpredictable sensitivity to a particular treatment among AML patients. It is plausible that advances in genome sequencing technology will allow for timely and economically feasible personalized therapy based on molecular profiling and *ex vivo* drug sensitivity and resistance testing [139,140,141]. It is tempting to speculate that such an ability to accurately tailor therapy might eventually abrogate the need for future “salvage therapy”.

In addition to AML clone biology characteristics that are postulated to contribute to therapy resistance and disease relapse, the bone marrow microenvironment has also been shown to affect therapeutic efficacy in patients with AML. A recent study found that AML cells can educate bone marrow-derived stromal cells to secrete Gas6, which fosters AML cell growth and chemoresistance via the receptor tyrosine kinase Axl [101]. Overexpression of CXCR4, a chemokine receptor, has been correlated with poor survival and its inhibition is the focus of several studies [142]. Combination therapy strategies that also target the microenvironment may be necessary in order to improve outcomes.

## 7. Conclusions

“Relapse” of AML is predicated on the concept of remission. The development of increasingly sensitive minimal or measurable residual disease (MRD) assays has demonstrated that current remission criteria, originally proposed in 1956, do not provide sensitive assessment of AML disease burden [136], as evidenced by the disconnect between the apparent success of current induction therapy in achieving complete remission in most patients and the stark reality of median overall survival times of less than two years [2]. Furthermore, studies evaluating remission status and subsequent relapse risk have shown traditional morphologic assessment is inferior when compared with newer methods such as flow cytometry [143,144,145] or PCR based detection of AML associated mutations [146] or gene over-expression [79,147,148]. Nevertheless, while it is clear that clinically evident relapsed AML represents an end-stage, advanced process that could potentially be detected at an earlier time utilizing a sensitive MRD assay, it is unclear how much more effective treatment at this earlier timepoint will be compared to treatment in the conventional salvage setting described herein [110,149,150]. It is likely that many patients experiencing AML “relapse” are in fact manifesting the clinical outgrowth of a refractory clone that has persisted despite apparently successful initial therapy [9]. We would argue, at least conceptually, that the main problem of AML relapse is not that we cannot adequately prevent or treat relapse, but rather that our apparently successful initial treatment was not as effective as we had hoped. Relapse is therefore not a sign that an initial successful treatment has now failed, but rather simply that it was not a successful treatment. It is in this context that the modest success of the aforementioned second-line therapies for relapsed AML should be judged and, in the absence of any obvious standard of care, we suggest that all patients with refractory or relapsed AML be offered a referral to an appropriate clinical trial whenever possible.
